# Nanodispersion of lutein with use of metastable polymorph for improved dissolution and oral absorption

**DOI:** 10.1016/j.pscia.2025.100067

**Published:** 2025-03-04

**Authors:** Kodai Ueno, Monami Sugihara, Tetsuya Matsushita, Kohei Yamada, Hideyuki Sato, Satomi Onoue

**Affiliations:** Laboratory of Biopharmacy, School of Pharmaceutical Sciences, University of Shizuoka, 52-1 Yada, Suruga-ku, Shizuoka, 422-8526, Japan

**Keywords:** Dissolution, Lutein, Metastable polymorph, Nanodispersion, Oral absorption, Photostability

## Abstract

Lutein (LT) is an attractive nutrient for eye health, although it has low water solubility and poor oral absorption. The present study aimed to develop a novel nanodispersion (ND) of LT using a metastable polymorph, offering improved oral absorption of LT. A metastable crystalline form of LT (LT-II) and hydroxypropyl cellulose were subjected to wet-milling followed by freeze-drying to obtain the ND of LT-II (ND/LT-II), and its physicochemical, photochemical, and pharmacokinetic properties of LT samples were evaluated. The mean particle size of LT-II in ND/LT-II was 354 ​nm, and there was no significant change in the crystalline form of LT-II, even after wet milling and freeze-drying. LT generated significant amounts of superoxide anions upon exposure to pseudo-sunlight (250 ​W/m^2^), indicating high photoreactivity. After irradiation with pseudo-sunlight (250 ​W/m^2^, 30 ​min), the percentages of LT remaining in the LT solution, amorphous LT, and ND/LT-II were 75, 79, and 92%, respectively. LT-II dissolved slightly faster than the stable crystalline form of LT (LT-I) in the dissolution media. ND/LT-II further improved the dissolution property of LT-II, and the dissolved amount of LT was 137- and 7.2-fold higher than that of LT-I and LT-II, respectively, at 2 ​h after dispersion in water. After administration of LT samples (100 ​mg-LT/kg), systemic exposure to LT in the LT-I and LT-II was negligible, whereas a marked improvement in oral absorption was observed in the ND/LT-II groups. Thus, applying ND technology to LT-II may improve oral absorption, and thus the nutrient function of LT.

## Abbreviations

ASD: Amorphous solid dispersion
AUC_0–12 ​h_: Area under the concentration versus time curve from 0 to 12 ​h
*C*_max_: Maximum concentration
DLS: Dynamic light scattering
DMSO: Dimethyl sulfoxide
HPC: Hydroxypropyl cellulose
HPLC: High-performance liquid chromatography
LDS: Laser diffraction scattering
LT: Lutein
NaPB: Sodium phosphate buffer
NBT: Nitroblue tetrazolium chloride
ND: Nanodispersion
PDI: Polydispersity index
QN: Quinine
ROS: Reactive oxygen species
SB: Sulisobenzone
SD: Standard deviation
SE: Standard error
SEM: Scanning electron microscope
*T*_max_: Time to reach maximum drug concentration
TEM: Transmission electron microscope
UV: Ultraviolet
XRPD: X-ray powder diffraction

## 1 Introduction

Lutein (LT) is a major xanthophyll carotenoid found in green leafy vegetables and marigold petals [1]. Several beneficial effects of LT on human health have been reported recently. For example, LT can help reduce the risk of several age-related eye diseases such as cataracts and macular degeneration [2,3]. Furthermore, high-dose LT intake can reduce the risk of cardiovascular diseases, cancers, and other diseases such as atherosclerosis [4]. Although LT have attractive functions, they cannot be synthesized in the human body and must be ingested [5]. If LT is used as a supplement, its nutritional function may not be fully realized owing to its low oral absorption potential (<10%) [6]. The low oral absorption of LT is due to its poor water solubility (0.732 ​μg/mL in water) [7], and improvement in its dissolution behavior may lead to enhanced oral absorption.

Various formulations have been developed to improve the dissolution of LT, including emulsions, micelles, liposomes, and amorphous solid dispersions [8,9]. Theoretically, chemicals are more prone to photodegradation in the liquid or amorphous state than in the crystalline state [10]. LT exhibits high UV-absorbing properties, and its conjugated double bonds can be readily oxidized and degraded by UV absorption [11]. Therefore, a solid formulation with high crystallinity would be desirable as an oral LT supplement. In our previous study, a metastable crystalline form of LT (LT-II) was obtained by the jet milling of a stable crystalline form (LT-I) in safflower oil [12]. LT-II showed a slightly higher dissolution rate (10.8 ​ng/min) than LT-I (2.0 ​ng/min) in water containing 1% Tween 80. However, this was not sufficiently high, and further improvements may be possible. To achieve both high crystallinity and better dissolution behavior, a nanodispersion (ND) system was developed using a wet-milling approach and applied to a number of chemicals [13,14]. In ND technology, active ingredients are pulverized to a nanometer size while maintaining their crystallinity, and then dispersed into hydrophilic polymers. These nanoparticles led to an increase in the surface area and a decrease in the diffusion layer thickness, consequently enhancing the dissolution rate of the chemicals [15]. Although applying the ND approach to LT-II may enhance its dissolution behavior and improve its oral absorption, its feasibility has not been fully clarified.

This study aimed to develop a new ND formulation of LT-II (ND/LT-II) with improved dissolution and oral absorption. ND/LT-II was prepared using a wet-milling system, followed by freeze-drying, and its physicochemical properties were evaluated in terms of morphology, particle size, crystallinity, and dissolution behavior. Photochemical characterization of ND/LT-II was conducted using a reactive oxygen species (ROS) assay and photostability tests. The pharmacokinetic behavior of LT was evaluated after the oral administration of ND/LT-II to rats. Based on the outcomes of physicochemical studies, taken together with biopharmaceutical characteristics, the combined use of the ND approach and a metastable polymorph led to the successful development of a promising dosage form of LT.

## 2 Materials and methods

### 2.1 Materials

LT-I and LT-II suspensions (LT-I and LT-II suspended in 20% safflower oil) were kindly supplied by AFC-HD AMS Life Science Co., Ltd. (Shizuoka, Japan). The LT quantitative standard (purity: 95.8%) was purchased from ChromaDex (Los Angeles, CA, USA). Low-viscosity hydroxypropyl cellulose (HPC-SSL, molecular weight: 15–30 ​kDa, viscosity of 2% solution at 20°C: 2–2.9 ​mPa·s) was purchased from Tokyo Chemical Industry Co., Ltd. (Tokyo, Japan). *n*-Hexane, Tween 80, dimethyl sulfoxide (DMSO), and anthracene were purchased from FUJIFILM Wako Pure Chemical Corporation (Osaka, Japan). All other chemicals used throughout the experiments were of special grade or high-performance liquid chromatography (HPLC) grade obtained from commercial sources.

### 2.2 Preparation of LT samples

#### 2.2.1 Purification of LT

*n*-Hexane (5 ​mL) was added to the LT-I or LT-II suspensions (5 ​mL) and mixed thoroughly. The mixture was centrifuged (1,250×*g*, 5 ​min) using a small centrifuge CT4D (Eppendorf Himac Technologies, Ibaraki, Japan), and the supernatant was removed. These operations were repeated 3 times, and the residue was dried in a vacuum desiccator for 2 days. The dried LT powder was used as LT-I or LT-II.

#### 2.2.2 Preparation of ND/LT-II

ND/LT-II was prepared using a wet-milling system using zirconia (zirconium oxide) balls based on our previous report with some modifications [16]. Briefly, 30 ​mg of LT-II was added to a 100 ​mL vessel of a rotation/revolution mixer (AR-100, Thinky Co., Ltd., Tokyo, Japan). Then, 2.5 ​g of Zirconia (zirconium oxide) balls with a diameter of 0.1 ​mm (YTZ ball with density of 3.7 ​kg/L, Nikkato Co., Ltd., Osaka, Japan) were added to the vessel, and the indicated volume of 1% HPC-SSL solution dissolved in distilled water was also added. The LT suspension was micronized by 3-step wet-milling with the following pulverizing conditions: pulverization process 1: 2,000 ​rpm for 1 ​min with 0.2 ​mL HPC-SSL solution; pulverization process 2: 2,000 ​rpm for 2 ​min with 0.5 ​mL HPC-SSL solution; dispersion process: 400 ​rpm for 1 ​min after the addition of 11.3 ​mL HPC-SSL solution. Subsequently, the suspension was freeze-dried (FD-1000; Tokyo Rikakikai, Tokyo, Japan).

### 2.3 Microscopic experiments

#### 2.3.1 Scanning electron microscope (SEM)

The LT samples were fixed on an aluminum sample holder with double-sided carbon tape and coated with platinum using an MSP-1S (Vacuum Device Inc., Ibaraki, Japan). Representative images of the LT samples were obtained by SEM (TM3030, Hitachi High-Tech Corporation, Tokyo, Japan).

#### 2.3.2 Transmission electron microscope (TEM)

Water-suspended ND/LT-II (5 ​μL) were placed on a mesh with a formbar membrane (Nisshin EM, Tokyo, Japan) and left to stand for 10 ​min. The samples were stained with a 25% EM Stainer and visualized using TEM (H-7600, Hitachi, Tokyo, Japan).

### 2.4 Particle size analysis

#### 2.4.1 Laser diffraction scattering (LDS)

LT samples were suspended in distilled water, and the particle size distribution of the LT particles was measured by an LDS method using Microtrac MT3000II (MicrotracBel, Osaka, Japan). The particle size distribution is expressed as the volume median diameter.

#### 2.4.2 Dynamic light scattering (DLS)

ND/LT-II were homogeneously dispersed in distilled water at 0.1 ​mg/mL. Size distribution and polydispersity index (PDI) were measured by DLS using a Zetasizer Nano ZS (Malvern Instruments, Malvern, UK).

### 2.5 X-ray powder diffraction (XRPD)

ND/LT-II was dispersed in distilled water and mixed thoroughly. The suspension was centrifuged (250,000×*g*, 20 ​min, 4°C) using a Hitachi ultracentrifuge (WX SERIES, Hitachi, Tokyo, Japan), and the supernatant was removed. The residue was dried in a vacuum desiccator for 3 ​h and used for the measurements. XRPD patterns of LT samples were collected using Mini Flex II (Rigaku Corporation, Tokyo, Japan) with Cu Ka radiation generated at 15 ​mA and 30 ​kV. Data were obtained from 3° to 40° (2*θ*) at a step size of 0.02° and scanning speed of 2°/min.

### 2.6 Photochemical characterization

#### 2.6.1 ROS assay

Singlet oxygen and superoxide anions generated from UV-irradiated LT, quinine (QN, positive control), and sulisobenzone (SB, negative control) at 20 ​μM were measured in accordance with the standard protocol of the ROS assay [17]. Briefly, 200 ​μL of samples containing the target compound under examination, *p*-nitrosodimethylaniline (50 ​μM), and imidazole (50 ​μM) in 20 ​mM NaPB (pH 7.4) were irradiated with simulated sunlight (250 ​W/m^2^, 25°C) using Atlas Suntest CPS+ (Atlas Material Technology LCC, Chicago, USA), which met the CIE85/1989 daylight simulation requirements. At the indicated time points (5, 10, 20, 30, 40, and 60 ​min), the UV absorption of the samples was measured at 440 ​nm using a SAFIRE (TECAN, Männedorf, Switzerland) to determine the singlet oxide. Samples (200 ​μL) containing the target compound under examination and NBT (50 ​μM) in 20 ​mM NaPB (pH 7.4) were irradiated with pseudo-sunlight under the same conditions as singlet oxygen. The reduction of NBT was evaluated by the increase in absorbance at 560 ​nm using SAFIRE to determine superoxide anions.

#### 2.6.2 Photostability test

Photostability tests were performed using an LT solution (LT dissolved in DMSO at a concentration of 1 ​mg/mL), amorphous LT, LT-I, LT-II, and ND/LT-II. Amorphous LT was prepared as follows: LT-I (14 ​mg) and HPC-SSL (86 ​mg) were dissolved in 1,4-dioxane (30 ​mL) and freeze-dried. LT samples were placed in a 24-well microplate (Asahi Glass, Tokyo, Japan) with 1 mg-LT/well, and irradiated with pseudo-sunlight (250 ​W/m^2^, 30 ​min, 25°C). After irradiation, the samples were dissolved in 2 ​mL of DMSO and the concentration of LT was determined using a Shimadzu Class-VP HPLC system (Shimadzu, Kyoto, Japan) equipped with an SIL-10ADvp auto-injector, LC-10ADvp solvent delivery pump, DGU-14A degasser, CTO-10Avp column oven, and an SPD-M10AVP UV-PDA detector with a detection wavelength of 445 ​nm. Inertsil ODS-4 (particle size: 3 ​μm; column size: 4.6 ​mm ​× ​150 ​mm, GL Sciences, Tokyo, Japan) was used, and the column temperature was maintained at 40°C. The mobile phase consisted of Milli-Q and methanol 4:96 (v/v) at a flow rate of 1.0 ​mL/min.

### 2.7 Dissolution test

A dissolution test of the LT samples (10 ​mg-LT) was carried out in 100 ​mL of water containing 1% Tween 80 ​at 37°C with constant stirring at 50 ​rpm using NTR-6100 ​A (Toyama Sangyo, Osaka, Japan). Samples (700 ​μL) were collected at the indicated time points (5, 10, 15, 30, 45, 60, 90, and 120 ​min) and filtered through a 0.02-μm Anotop filter (Whatman, Maidstone, UK). The filtered samples (500 ​μL) were dried under nitrogen gas and the residues were dissolved in 100 ​μL of methanol. The concentration of LT was measured using HPLC, as described in Section 2.6.2.

### 2.8 Pharmacokinetic study

#### 2.8.1 Animals

Male Sprague Dawley (SD) rats with 240±46 ​g body weight (Japan SLC, Shizuoka, Japan) were housed with free access to food and water under a 12-h dark/light cycle at a controlled temperature (24±1°C) and humidity (55±5% RH). The rats were fasted for 12 ​h before the oral administration of LT samples. All procedures involved in this study were conducted in accordance with the guidelines approved by the Institutional Animal Care and Ethical Committee of the University of Shizuoka (Approval No. 236598).

#### 2.8.2 Pharmacokinetic study

LT samples (100 ​mg-LT/kg body weight) were orally administered to fasted rats. Blood samples were collected from the tail vein into heparinized tubes at the indicated time points (1, 2, 4, 6, 8, 10, and 12 ​h) and centrifuged at 6,000×*g* for 10 ​min to obtain plasma samples. The plasma samples (100 ​μL) were deproteinized by the addition of methanol (300 ​μL) containing anthracene (150 ​ng/mL) as an internal standard, and the samples were centrifuged (10,000×*g*, 10 ​min, 4°C). The supernatants (300 ​μL) were dried under nitrogen gas, and the residues were dissolved in methanol (100 ​μL) and filtered through a 0.2-μm filter. The LT concentration was measured using HPLC, as described in Section 2.6.2. The area under the concentration versus time curve from 0 to 12 ​h (AUC_0–12h_) was calculated using the GraphPad Prism 4.03 (GraphPad Software, San Diego, CA, USA).

### 2.9 Data analysis

Data were presented as mean±standard deviation (mean±SD) or mean±standard error (mean±SE). One-way analysis of variance (ANOVA) followed by Tukey's multiple comparison test was used for statistical comparisons. Statistical significance was set at *p* ​< ​0.05.

## 3 Results and discussion

### 3.1 Physicochemical properties

**Fig. 1:**
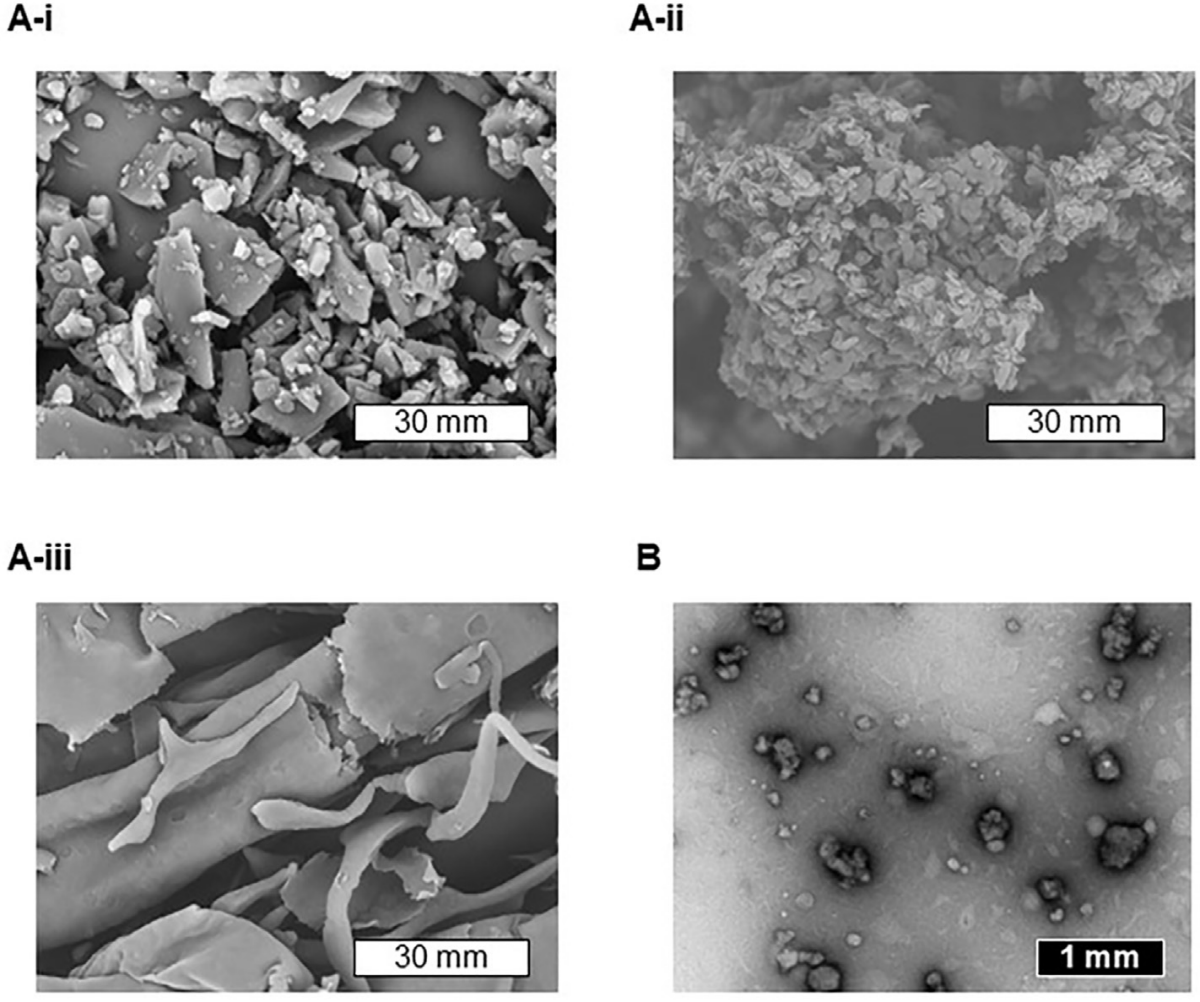
Micrographic images of LT samples. (A) SEM images of (i) LT-I, (ii) LT-II, and (iii) ND/LT-II. White bars represent 30 ​μm. (B) TEM image of ND/LT-II. Black bar represents 1 ​μm.

**Fig. 2:**
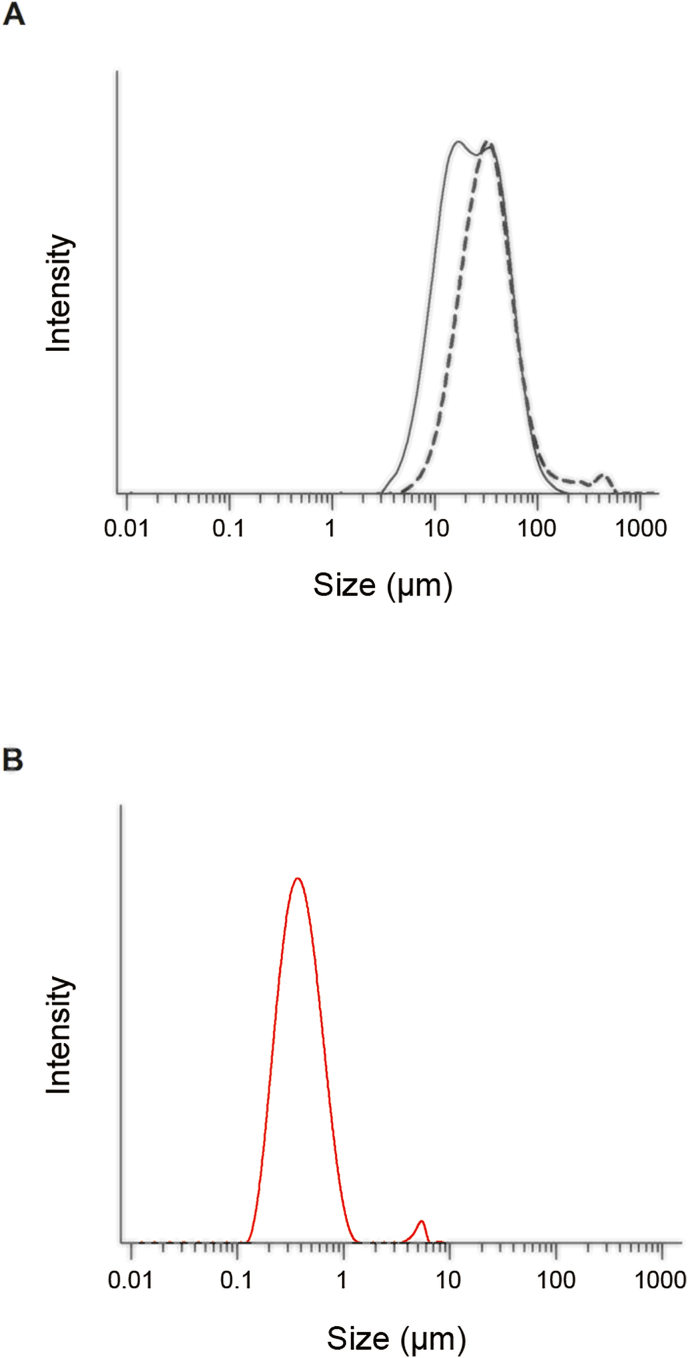
Particle size distribution of LT samples. (A) LDS analyses of LT-I (dashed line) and LT-II (solid line). (B) DLS analysis of ND/LT-II dispersed in water.

**Fig. 3:**
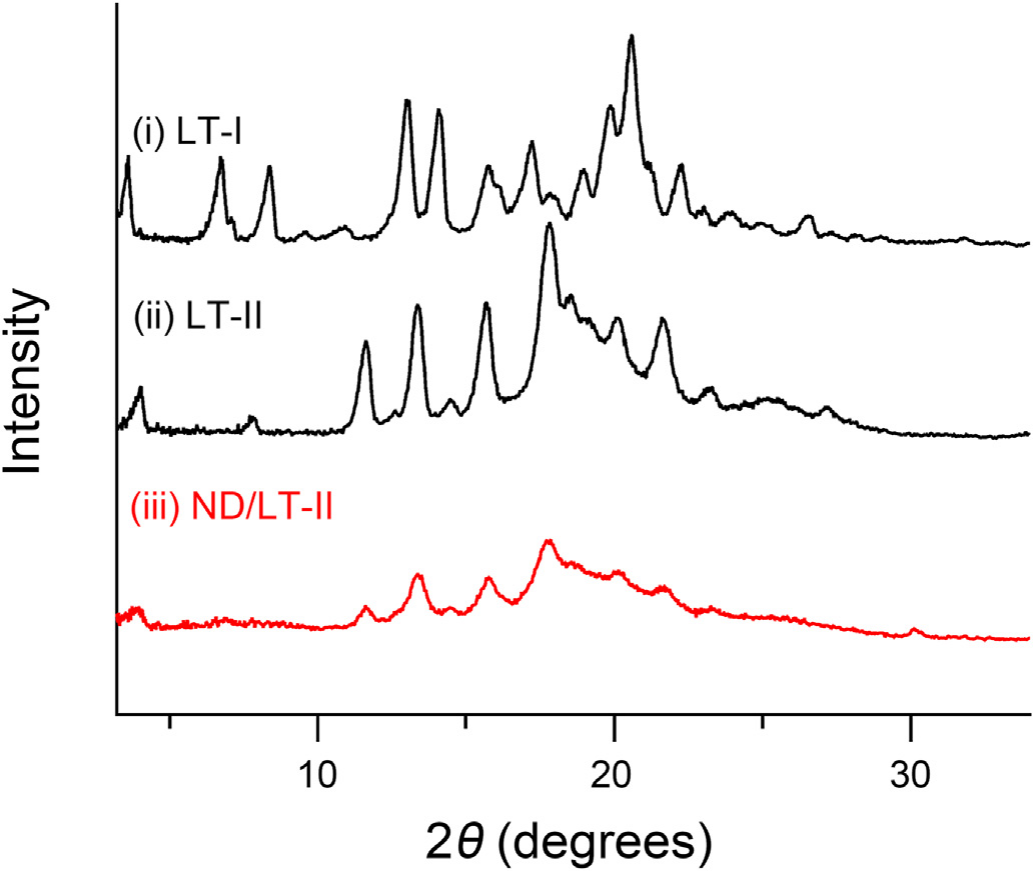
XRPD patterns of (i) LT-I, (ii) LT-II, and (iii) ND/LT-II.

Theoretically, a metastable polymorph has a higher energy state and better dissolution properties than a stable polymorph [18]. In our previous studies, LT-II was identified as a new polymorph with a higher dissolution rate than LT-I [12]. To further enhance the dissolution of LT, ND/LT-II was prepared via wet milling and lyophilization. According to SEM images, LT-I was coarse and irregular in shape, LT-II had a scale-like morphology, and ND/LT-II had a thin sheet-like structure (Fig. 1A). Irregularly shaped nanoparticles were observed in the TEM images of water-dispersed ND/LT-II (Fig. 1B). LDS and DLS analyses were conducted to determine the particle sizes of the LT samples. The average particle sizes of LT-I and LT-II were calculated as 42.0 and 26.7 ​μm, respectively, using LDS analysis (Fig. 2A). In contrast, the DLS data of water-dispersed ND/LT-II demonstrated the formation of uniformly nanosized particles with a mean particle size of 354 ​nm and a PDI of 0.23 (Fig. 2B). The XRPD pattern of ND/LT-II showed a peak identical to that of LT-II (Fig. 3), suggesting that the wet milling and lyophilization processes had no major impact on the crystallinity of LT-II.

The wet-milling process often causes decomposition, aggregation, and crystal transition of compounds owing to its high-energy load [13]. Some chemicals form hydrates after wet-milling [19]. LT-II was nanomized to a mean particle size of 354 ​nm without major decomposition and underwent aggregation by wet milling, and the XRPD patterns did not suggest any changes in crystallinity or the formation of hydrates in LT-II after wet milling. HPC, an amphiphilic polymer, was used to prepare the ND/LT-II. It has been reported that such amphiphilic polymers provide high dispersion stability to nanoparticles and suppress crystal transitions by interacting with chemicals [20]; therefore, the HPC polymer may have contributed to the higher stability of ND/LT-II. The inner LT-II nanoparticles in ND/LT-II could be easily released from the HPC polymers in water and might improve the dissolution properties of LT.

### 3.2 Photochemical properties

**Fig. 4:**
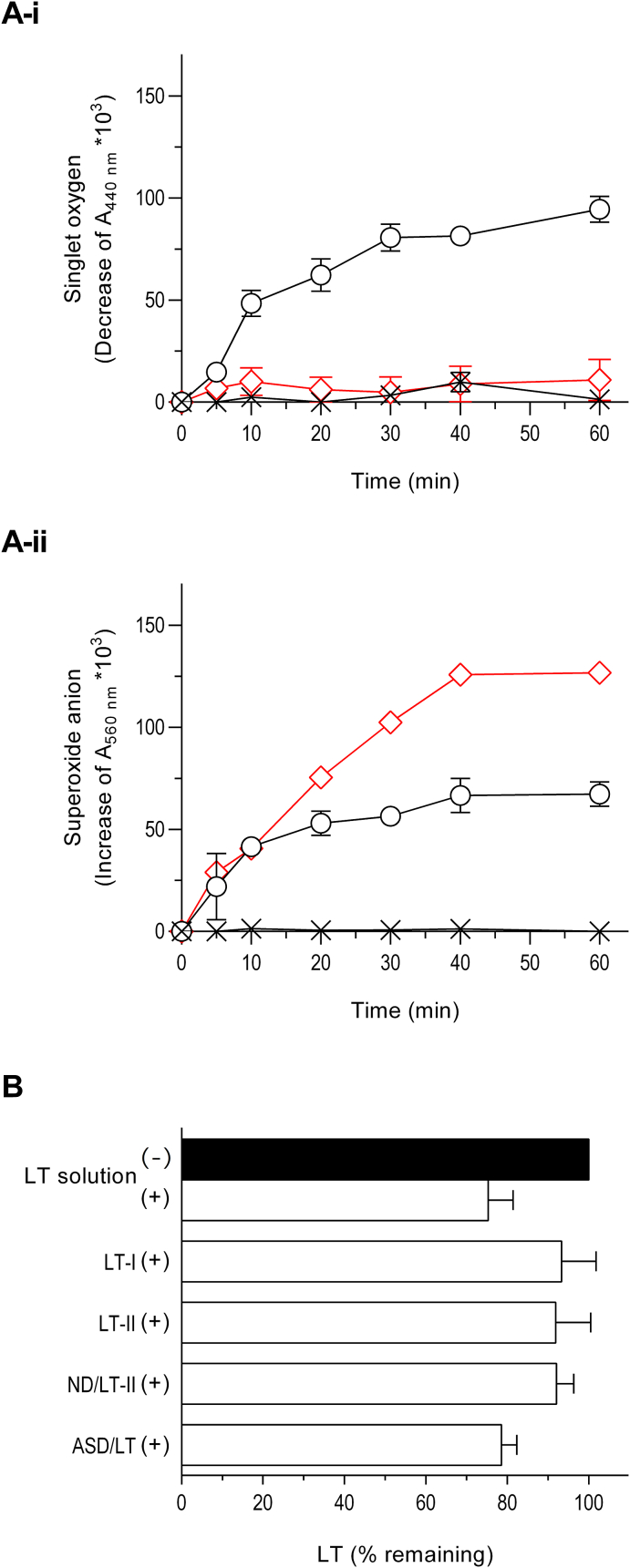
Photochemical properties of LT samples. Generation of ROS including singlet oxygen (A-i) and the superoxide anion (A-ii) from LT (20 ​μM), QN (20 ​μM), and SB (20 ​μM) exposed to simulated sunlight (250 ​W/m^2^). ◇, LT; ○, QN (positive control); and ​× ​, SB (negative control). Data represent mean ​± ​SD of 3 experiments. (B) Photostability of LT samples on exposure to simulated sunlight (250 ​W/m^2^, 30 ​min). (−), Non-irradiation; and (+), irradiation. Data represent mean±SD of 3 experiments.

LT is a highly UV-absorbing compound in the blue light region (400–500 ​nm wavelength) [21], and its photostability during storage and manufacturing is a common concern [22]. Some chemicals undergo photochemical reactions mediated by ROS generation; therefore, the amount of ROS generated after photoirradiation is an indicator of the photoreactivity of the chemicals [23]. A ROS assay was performed to assess the photoreactivity of LT (Fig. 4A). Photoirradiated LT yielded superoxide anions in a time-dependent manner. This level generated from LT at a concentration as low as 20 ​μM were much higher than the criteria (⊿A_560nm_ ​× ​10^3^:25) for the ROS assay at a concentration of 200 ​μM; thus, LT could be judged as photoreactive [24].

Photostability tests were conducted using a solar simulator to assess the risk of photodegradation of ND/LT-II. For comparison, various LT samples were exposed to simulated sunlight (250 ​W/m^2^, 30 ​min) (Fig. 4B). The remaining LT levels after photoirradiation were calculated to be 75% for the LT solution, 79% for amorphous LT, and above 90% for LT-I, LT-II, and ND/LT-II.

Photoirradiated LT yielded superoxide anions and underwent marked photodegradation of LT in the LT solution and amorphous LT, whereas ND/LT-II, LT-I and LT-II did not show significant photodegradation. From the XRPD analyses, ND/LT-II showed identical peak patterns to those of LT-II, and the high crystallinity of ND/LT-II may have contributed to the high photostability of LT. In addition to the ND approach, the amorphous solid dispersion (ASD) approach is often used to improve the dissolution properties of poorly soluble chemicals [25,26]. As LT showed significant photodegradation in the amorphous state, the ND formulation was more suitable for LT than the ASD formulation. Carotenoids, including LT, have been reported to undergo photodegradation after light exposure, resulting in a decrease in free radical scavenging activity [27]. Although ND/LT-II showed a higher photostability than the LT solution and amorphous LT, slight photodegradation was still observed in the photostability test, possibly leading to a slight loss of biological activity. Therefore, protection from light is preferred for the ND/LT-II.

### 3.3 Dissolution behaviors

**Fig. 5:**
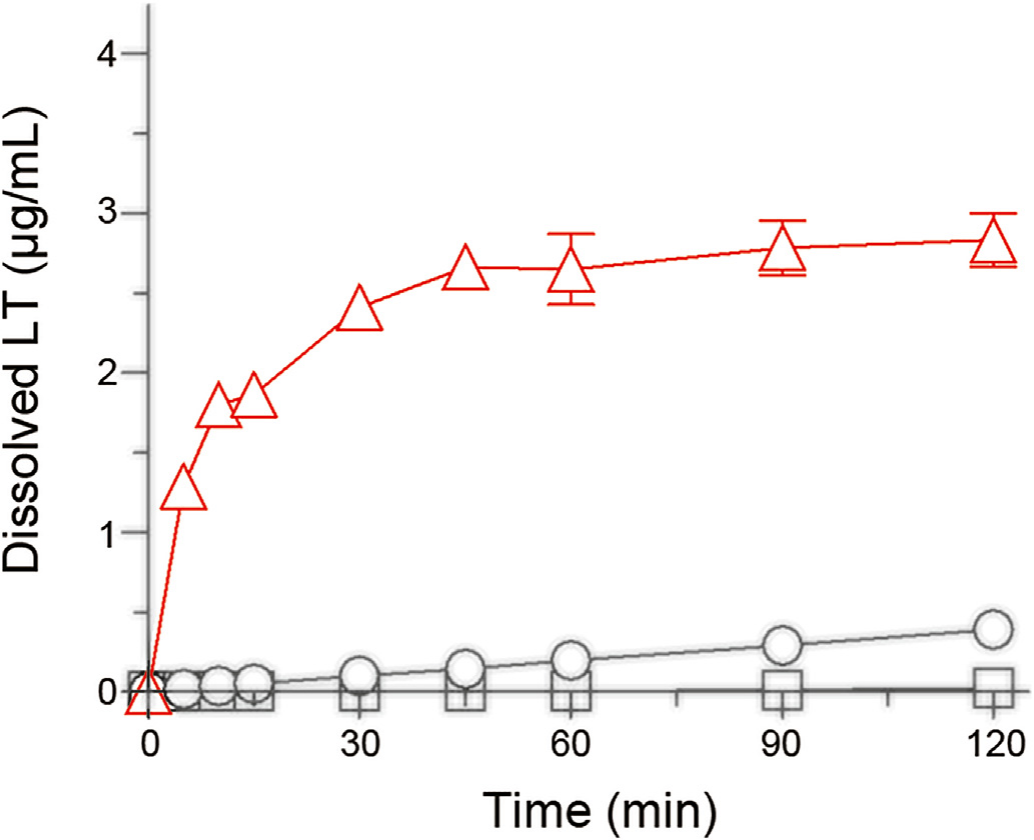
Dissolution profiles of LT samples in water containing 1% (v/v) Tween 80. □, LT-I; ○, LT-II; and △, ND/LT-II. Data represent mean±SD of 3 experiments.

ND/LT-II were dispersed in water as nanoparticles with an average particle size of 354 ​nm. Such nanoparticles increase the surface area and decrease the diffusion layer thickness, consequently leading to an improved chemical dissolution behavior [15]. To clarify the possible improvements in the dissolution properties of ND/LT-II, a dissolution test was conducted on the LT samples (Fig. 5). Because LT is mainly absorbed in the small intestine [28], an aqueous solution containing 1% Tween 80 was used as the simulated intestinal fluid for the dissolution test [29]. The dissolution of LT-I was negligible throughout this study, whereas LT-II showed a gradual increase in dissolved LT levels and slightly faster dissolution. These observations are consistent with our previous data, showing that LT-II has better dissolution properties than LT-I. ND/LT-II showed a significant improvement in the dissolution behavior of LT, as evidenced by the 140- and 7.2-fold higher dissolved amounts of LT compared to LT-I and LT-II at 2 ​h after dispersion in water, respectively. Thus, the strategic use of the ND approach for LT-II may further enhance its dissolution properties.

LT-II dissolved faster than LT-I, and the ND formulation of LT-II was prepared with the remaining crystalline form of LT-II, consequently leading to a marked improvement in the dissolution behavior of LT. Thus, applying the ND approach to chemicals with a more soluble polymorph may be effective in improving the dissolution properties of poorly soluble chemicals. SEM and TEM analyses of ND/LT-II suggested that the LT nanoparticles were uniformly dispersed in the HPC polymer, and the inner LT nanoparticles were easily released from the HPC polymer in water. The high re-dispersibility of the LT-II nanoparticles may have also contributed to the improved dissolution properties of ND/LT-II. As the oral absorption of LT is limited owing to its poor dissolution behavior in the small intestine, the enhanced dissolution behavior of LT mediated by the ND approach may lead to improved oral absorption.

### 3.4 Pharmacokinetic profiles

**Fig. 6:**
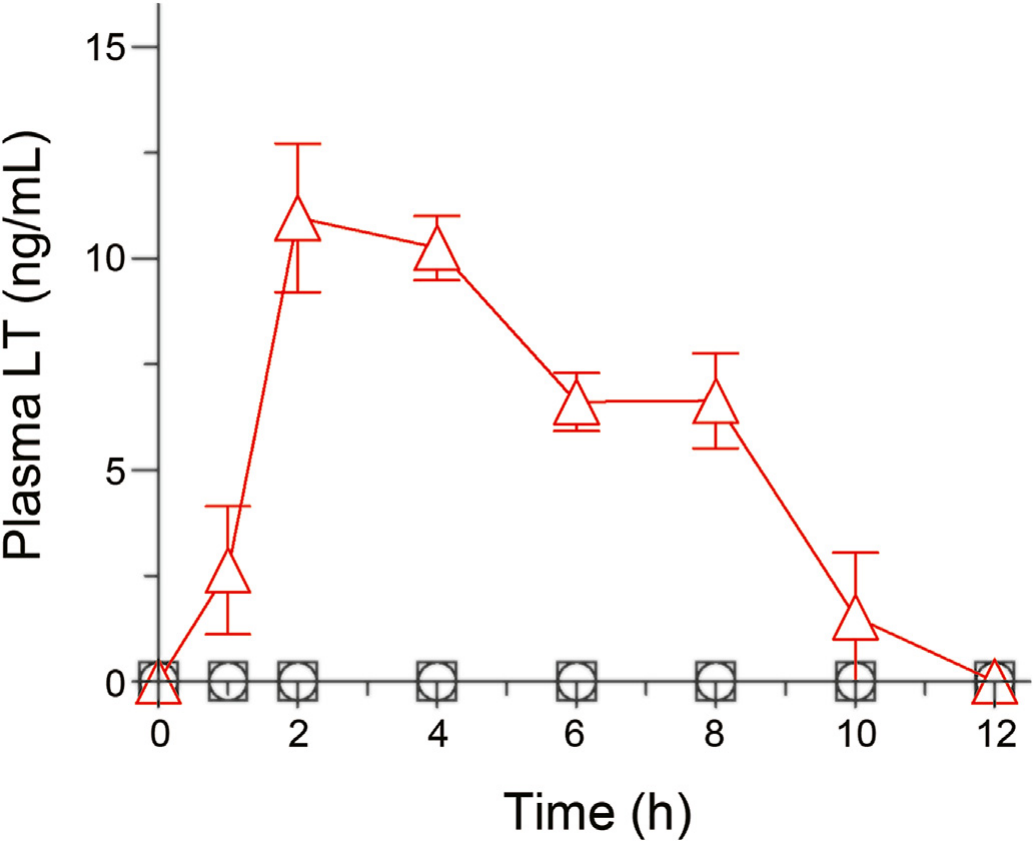
Plasma concentration profiles of LT samples after oral administration at 100 ​mg-LT/kg in rats. □, LT-I; ○, LT-II; and △, ND/LT-II. Data represent mean±SE of 4 experiments.

The observations of the improved dissolution behavior of ND/LT-II prompted us to clarify the possible improvement in the oral absorption of LT. Therefore, a pharmacokinetic study after the oral administration of ND/LT-II was carried out in rats. After the oral administration of the LT samples, the plasma concentration of LT was determined by HPLC analysis at various time points (Fig. 6). Relevant pharmacokinetic parameters, including *C*_max_, *T*_max_, and AUC_0–12h_ were calculated and are listed in Table 1. After LT-I and LT-II administration, systemic exposure to LT was negligible, and pharmacokinetic parameters were unavailable. These results suggest that LT-II did not lead to the observed improvement in the oral absorption of LT, even though LT-II showed slightly faster dissolution than LT-I. In contrast, ND/LT-II showed a significant improvement in the pharmacokinetic behavior of LT compared to LT-I and LT-II. These results are in agreement with the results of the dissolution test, demonstrating that the ND approach led to a significant improvement in the dissolution behavior of LT-II. The *C*_max_ and AUC_0–12h_ values of ND/LT-II were calculated as 11.5 ​ng/mL and 69.1 ​ng·h/mL, respectively. In our previous study, after administration of LT-I at doses as high as 300 ​mg/kg, the *C*_max_ value was calculated to be only 2.1 ​ng/mL [21]. This suggests that ND/LT-II can achieve higher blood LT levels than LT-I at a much lower dose. The improved oral absorption of LT by ND/LT-II could improve not only the nutrient functions of LT but also the convenience of using LT products by reducing the amount that needs to be taken.

**Table 1:** Pharmacokinetic parameters of LT samples after oral administration in rats.

Samples	*C*_max_ (ng/mL)	AUC_0–12h_ (ng·h/mL)	*T*_max_ (h)
LT-I	N.D.	–	–
LT-II	N.D.	–	–
ND/LT-II	11.5 ​± ​1.4	69.1 ​± ​9.4	3.0 ​± ​0.6

N.D., Not detected. Data represent mean±SE of 4 experiments.

LT is absorbed by epithelial cells in the upper gastrointestinal tract by passive diffusion and transferred into the blood via the lymph stream [28]. For chemicals with low solubility and lipophilic properties, such as LT, passive diffusion is often rate-limited by dissolution in the gastrointestinal tract, and these chemicals may not be completely absorbed within the residence time in the gastrointestinal tract because of their low absorption rate. ND/LT-II showed a significantly improved LT dissolution rate. In addition, nanoparticles generally have strong mucoadhesive properties and are retained in the small intestinal mucosa [30]. ND/LT-II might have extended the retention time of LT in the gastrointestinal tract, thereby significantly improving the oral absorption of LT. From the pharmacokinetic profile of ND/LT-II, the elimination of LT from the blood was rapid, as evidenced by blood LT levels below the detection limit at 12 ​h after the administration of ND/LT-II. For the long-lasting nutrient function of LT, ND/LT-II should be administered every 12 ​h to maintain high blood LT levels.

LT mainly demonstrate nutrient functions in the eyes; therefore, the distribution of LT to the ocular tissue after oral intake is important. Our previous study showed that improved absorption of orally could leads to increased LT accumulation in the eyes of rats [21]. This is because LT is highly distributed and persistently retained in the eye in vivo owing to the presence of LT-binding proteins in the retina [31]. Thus, ND/LT-II may achieve higher LT distribution in the ocular tissue by improving systemic exposure to LT, resulting in enhanced nutrient functions in the eyes.

## 4 Limitations

In this study, the newly developed NC/LT-II exhibited preferable biopharmaceutical and nutraceutical properties; however, it is still unclear whether there is a gradual transition in these properties after long-term storage. If there are significant changes in the dissolution behavior of NC/LT-II owing to moisture absorption and/or crystal transition during storage, careful consideration should be given when setting the appropriate packaging forms and storage conditions. In the pharmacokinetic study, oral absorption of LT samples was evaluated only in rats. Because rats are gallbladder-free animals, they have higher bile acid concentrations in the small intestine than humans [32]. Therefore, there may be species differences in the pharmacokinetic behavior of NC/LT-II, especially in the absorption kinetics and oral bioavailability, between rats and humans. To better understand the pharmacokinetic properties of ND/LT-II, it is necessary to examine the gallbladder in humans or other appropriate experimental animals.

## 5 Conclusion

In conclusion, an ND formulation of LT was designed using a metastable polymorph. ND formulations of LT-II were successfully prepared by a wet-mill process and showed significant improvements in both the dissolution and pharmacokinetic behavior of LT compared to LT-I and LT-II. Based on these observations, the combined use of ND and a metastable polymorph could be a viable strategy to improve the biopharmaceutical and nutraceutical properties of LT.

## CRediT authorship contribution statement

**Kodai Ueno:** Writing – original draft, Investigation, Conceptualization. **Monami Sugihara:** Methodology, Investigation. **Tetsuya Matsushita:** Methodology, Investigation, Data curation. **Kohei Yamada:** Writing – review & editing, Investigation. **Hideyuki Sato:** Methodology, Investigation. **Satomi Onoue:** Writing – review & editing, Methodology, Funding acquisition, Conceptualization.

## Data availability statement

The data supporting the findings of this study are available from the corresponding author upon request.

## Ethics approval

All animal testing procedures were conducted in accordance with the guidelines approved by the Institutional Animal Care and Ethical Committee of the University of Shizuoka (Approval No. 236598).

## Funding

This work was supported in part by JSPS KAKENHI [Grants-in-Aid for Scientific Research (C) (no. 24K09917: S.O. and no. 20K07180: H.S.]).

## Declaration of interests

The authors declare that they have no known competing financial interests or personal relationships that could have appeared to influence the work reported in this paper.
